# Neurophysiology of slip sensation and grip reaction: insights for hand prosthesis control of slippage

**DOI:** 10.1152/jn.00087.2021

**Published:** 2021-07-07

**Authors:** Andrea Zangrandi, Marco D’Alonzo, Christian Cipriani, Giovanni Di Pino

**Affiliations:** 1 Research Unit of Neurophysiology and Neuroengineering of Human-Technology Interaction (NeXTlab), Università Campus Bio-Medico di Roma, Rome, Italy; 2 The BioRobotics Institute, Scuola Superiore Sant’Anna, Pisa, Italy; 3 Department of Excellence in Robotics & A.I., Scuola Superiore Sant’Anna, Pisa, Italy

**Keywords:** physiology of slippage, reflexes, sensorimotor control loop, touch, upper limb prostheses

## Abstract

Sensory feedback is pivotal for a proficient dexterity of the hand. By modulating the grip force in function of the quick and not completely predictable change of the load force, grabbed objects are prevented to slip from the hand. Slippage control is an enabling achievement to all manipulation abilities. However, in hand prosthetics, the performance of even the most innovative research solutions proposed so far to control slippage remain distant from the human physiology. Indeed, slippage control involves parallel and compensatory activation of multiple mechanoceptors, spinal and supraspinal reflexes, and higher-order voluntary behavioral adjustments. In this work, we reviewed the literature on physiological correlates of slippage to propose a three-phases model for the slip sensation and reaction. Furthermore, we discuss the main strategies employed so far in the research studies that tried to restore slippage control in amputees. In the light of the proposed three-phase slippage model and from the weaknesses of already implemented solutions, we proposed several physiology-inspired solutions for slippage control to be implemented in the future hand prostheses. Understanding the physiological basis of slip detection and perception and implementing them in novel hand feedback system would make prosthesis manipulation more efficient and would boost its perceived naturalness, fostering the sense of agency for the hand movements.

## Introduction

Skilled dexterous manipulation requires precise modulation of muscular outputs based on relevant sensory cues (1, 2). In particular, sensory feedback is fundamental to compensate for unpredicted events during the interaction with the environment.

**Figure 1:**
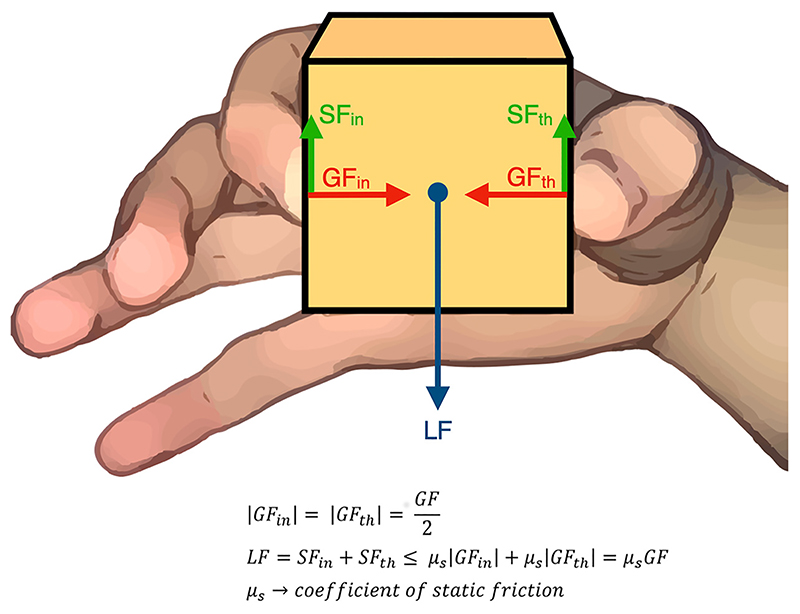
Visual representation of the grip force (GF) (i.e. the sum in absolute value of the normal forces generated by the digits on the grip surfaces: GF_in_ and GF_th_) and the forces tangential to the grip surface (load and shear forces, LF, SF_in_ and SF_th_) in the pinch grasp of an object between the pads of the thumb and the index finger. LF is the driving force; whereas SF is the global reaction force (sum of the tangential components of the forces generated by digits: SF_in_ and SF_th_) that allows to maintain the object stable on the hand. When the object is at equilibrium the sum of SF and LF is equal to 0. The maximum sustainable LF is equal to the coefficient of static friction (μ_s_) multiplied by the GF.

To obtain an efficient object manipulation, the force normal to the grip surface (grip force; GF) has to be timely modulated in relation to changes of the driving force tangential to the grip surface (3–5). The total reaction force, being equal to the driving force but with opposite sign, allows to maintain stability of a handheld object (Fig. 1). Not all the previous studies on the topic agree on the same taxonomy, and sometime the terms are switched. As we focus on avoiding and compensating slippage, naming the driving force due to the weight of the object load force (LF) and shear force (SF) the reaction one seems to be appropriate. The stability of a handheld object depends on such GF and on the characteristics of the interface between the two surfaces in contact (e.g., presence of asperities or lubricant on surfaces). The static friction, determining adhesion between surfaces, is due to electrostatic and/or Van der Waals and hydrogen bonding forces present at micrometer scale. The maximum LF sustainable, the least force required to move the object, is equal to GF multiplied by the coefficient of static friction (μ_*s*_) between the skin and the object. Therefore, to prevent the occurrence of slip, the GF/LF ratio must exceed a minimal value determined by the coefficient of static (i.e., the inverse of static friction) (6). If the LF increases, the fingertip is deformed until GF/LF ratio reaches the breakaway limit (i.e., below the inverse of coefficient of static friction), leading to the beginning of slip, that is, a relative motion between the digits and the object surface.

When object begins to move, the SF due to friction decreases because it becomes equal to GF multiplied by the coefficient of dynamic friction, which is lower than the static friction one. At this point, the SF is not able to compensate the LF and the object starts to accelerate. Applying an extremely high GF would increase the maximum sustainable LF and certainly avoid slippage, but this would also determine a useless effort and implies the risk of damaging fragile objects such as a champagne glass. For such reasons, subjects typically adjust the GF before voluntary self-initiated movements, considering previous liftings with the object or visually estimating its physical proprieties (i.e., expected weight, surface friction, etc.) and the environmental conditions (7). Moreover, they anticipate predictable variations in the LF adjusting the GF in advance, for example increasing the GF while someone is pouring water within a handheld cup (8,9).

According to the internal models theory, subjects maintain GF 10% to 40% above the minimum force needed to oppose the LF (so-called “safety margin”) to make manipulation of objects both safe and effortless (10–16). The safety margins ensure an efficient manipulation because if GF is too weak, slippage occurs and the object may drop; instead, an excessive GF force may cause unnecessary muscular fatigue or damage the object. The GF/LF ratio critically depends on the parameters of the hand-object interaction. The pinch grasp (showed in the Fig. 1) is the one typically tested in manipulation studies, but other strategies are frequently used in daily life, as when the palm is employed in heavy and bimanual manipulation. When two or more areas with a different coefficient of friction are used, the GF/LF ratio is scaled mainly on the basis of the local friction at each individual area (17–19), thus the applied GF varies to ensure an optimal safety margin depending on the grasping configuration (20). For instance, the coefficient of friction of the palm is higher than the one of the fingertips, which are involved in precise manipulation (20). Accordingly, the CNS adapts the control strategies considering the task to be performed. For example, a lower safety margin is found during static object manipulation tasks (e.g., when exerting forces over an external support), as compared with free object manipulation tasks (during which the weight and inertia of the object needs to be considered to adjust the GF) (21, 22). The safety margin also provides a time window in which a prompt reaction may prevent incipient slippage. Indeed, an increase of the safety margin associated with an excessive GF is the result of the disruption of sensory information and/or sensory processing after digital anesthesia (2, 23), when wearing gloves (24), with age (25), and in multiple sclerosis or stroke (26–30).

In principle, object properties and load are predicted. Once the object is grasped and lifted, the afferent information relative to load is fed back to the nervous system, that rapidly and dynamically re-adapt the GF. When, in natural (1, 2) or experimental circumstances (31), perturbations completely overcome the safety margin, there is a transition from stuck to slip and several automatic and voluntary GF responses can be observed. Thousands of parallel afferents from mechanoreceptors in the skin, tendons and muscles of the hand and forearm provide to the CNS essential information on the properties of the touched surface, allowing the (largely unconscious) regulation of the GF, via both spinal and supraspinal sensorimotor systems. This complex mechanism of control is abolished in case of upper limb amputations, that besides of the obvious loss of the hand effector, severely impair both motor and sensory control systems, further cause of the related severe functional disability. Several attempts have been made to reproduce in upper limb pros-theses the lost abilities of the hand, yet this goal is difficult to achieve because, up-to-date technological solutions have not reached the complexity of the human physiology. The manipulative abilities of the commercial/clinical prosthetic devices are limited for several reasons (32): *1)* only few hand configurations can be reproduced; *2)* movements are controlled trough the contraction of residual muscles originally not involved in the action (e.g., biceps contraction to close the prosthetic hand); *3)* most importantly, such devices are not able to provide the richness of the feedback about the physical interaction with the environment once provided by the lost hand (33). The lack of tactile feedback about the prosthesis status requires the user to rely mostly on visual and auditory information to perform the task, causing a significant cognitive effort that is among the causes for the abandonment of active prosthetic hands (34).

In the latest years, several invasive and noninvasive attempts have been made in research laboratories to resemble the human hand sensory performance. These proved the potential to achieve an improved motor control (35–47), a more natural sensory feedback (44–46), and proprioception (48, 49). Recent evidence showed that the real-time feedback on the slippage is an important feature that the prosthesis has to integrate (47) to allow for routine activities safely. Indeed, previous studies demonstrated that grasp control is more efficient when based on information relative to the slip of a manipulated object, rather than on forces exerted by the fingers (e.g., grasping force) (50–52). Commercial, and mostly of research, hand prostheses prevent slippage by relying on automatic grip controllers based on interaction forces with the grasped object and bypassing the user control (53, 54). Stabilization of the object in the hand is achieved relying only on the physical/mechanical features of the materials of the prosthesis and rarely on automatic control algorithms, while no tactile feedback is provided to the user. However, algorithms hardly discern if a subtle muscle activity is due to a grip reflex or to a control command generated intentionally by the user (52). In these cases, providing the user with a sensory feedback is the only way to avoid dropping the grasped object. The different research solutions proposed so far to counteract slippage conveying feedback from the prosthesis to the user remained distant from the human physiology, probably because the physiology of slippage is complex, still not completely elucidated, and difficult to be implemented in prosthetic arms. Understanding the physiological basis of slip perception is fundamental to design and develop an afferent biologic-inspired feedback system, which aims not only to be efficient, but also to be more natural for the user and, hence, to foster the embodiment of the device.

Previous papers described the tactile control of object manipulation in humans focusing on tactile events that define all its phases, for example, reaching, loading, lifting, holding, etc. [see Johansson and Flanagan for a comprehensive review (4, 5)]. However, the physiological events that define slippage have been not extensively addressed. This work is specifically aimed at reviewing the neurophysiological basis of slippage, with the perspective of their possible implementation in hand prosthetics to improve their manipulation abilities and naturalness. We analyzed the literature on physiologic correlates of slippage and of the reaction to it and propose a three-phases (i.e., stuck, partial slip, and full slip phases) model for the slip perception. The model we employed has been built starting from a stable condition of secure grip (i.e., the “stuck phase” corresponding to the “holding phase” of Refs. 4 and 5), but with the different outcome of object slippage due to a perturbation, instead of object safety replaced. Lastly, we discuss strategies to provide slippage feedback, focusing on prosthetic solutions. In particular, the physiology-based solutions were identified and discussed in the light of the proposed three-phase model and of the physiological slip responses. In appendix, a quick overview on the platforms employed in neurophysiologic studies on the characteristics of the slippage (e.g., interaction forces, fingertip deformation, etc.) helps understanding how slippage can be investigated.

## How Does Slippage Occur?

**Figure 2:**
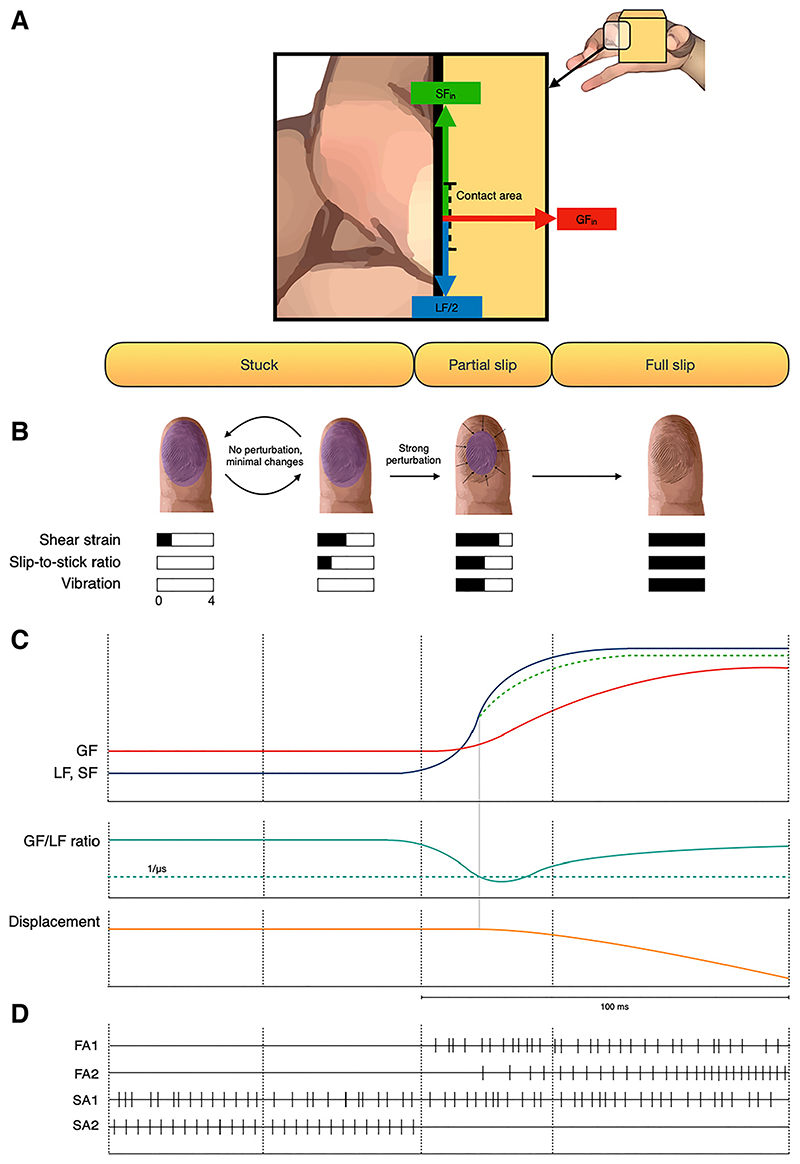
*A:* visual representation of the interface between the index finger and the object. The contact area (dotted line), the grip force (red arrow), and the shear force (green arrow) generated by the index finger are showed. In the pinch grip, the shear force generated by the index finger balances half of the load force (LF/2). *B:* schematic quantification of shearing strain of the fingertip (shear strain), the ratio of the slipping area to the entire contact area (slip-to-stick ratio) and vibration, represented on a 5-points scale during each of the three slippage phases. The contact area between the fingertip and the object is highlighted in violet. The slip-to-stick ratio falls progressively from 0 (no slip) to 1 (full slip) as partial slip develops over time. *C:* relation between the grip force (GF, in red) shear and load force (SF and LF, in green and blue) during each of the three slippage phases. In the “stuck phase,” the LF and SF have the same module whereas the object is kept safely on the hand. In the “partial slip phase,” a sudden increase of the LF is succeeded by a reactive increase of GF force to compensate the perturbation and, hence, increase the maximum sustainable LF. In the “full slip phase,” when the object slides over the finger pad because, the LF is higher than the SF. The ratio between the GF and the LF is showed in green, the displacement of the object relative to the fingers (in orange) happens when that ratio value goes below the inverse of the coefficient of static friction (dotted green line). *D:* discharges of fast and slow afferent fibers during each of the three slippage phases.

Somatosensory feedback starts from the mechanoreceptors of the hand and provides the sense of touch, pressure, vibration, and cutaneous stretch to the central nervous system (CNS). Four main classes of tactile units are present in the glabrous skin of the human hand, based on their microneurographic response (55, 56): rapidly adapting (RA1 and RA2) or slowly adapting (SA1 and SA2) superficial and profound afferents (57, 58). Different units correspond to a specific tactile receptor: SA1 afferents end in Merkel cells, RA1 end in Meissner corpuscles, RA2 end in Pacinian corpuscles, and SA2 afferents terminate in Ruffini corpuscles (59). Each type of afferent has a different function in slippage detection (Table 1). When a grasped object slips off the hand, a fast transition from stick to slip occurs with complex dynamics that can be summarized in three phases (“stuck phase,” “partial slip,” and “full slip”) (Fig. 2) (62–64). During all of these phases, the relative motion between the surface of the object and the fingers produces vibrations and patterns of skin deformation, such as stretch and indentation, that are detected by the different populations of mechanoreceptors that have specific contributions depending on the phase (Table 1).

### Stuck Phase

In the first phase, named “stuck phase,” the SF only slightly changes, without producing any macroscopic displacement of the contacting surfaces. This is a static phase where a shear strain is applied to the finger pad without the occurrence of relative slip. In the absence of relative motion between skin and an object, only slow adapting units respond in a continuous way. SA1 fibers respond with a sustained discharge that is linearly related to indentation depth (65), whereas SA2 fibers efficiently signal the horizontal skin stretch (59). In addition, silence of FA1 afferents indirectly suggests that changes in tangential forces do not occur. An important parameter that seems to affect the grip force strategy is the hydration of the skin (i.e., moisture level), as the more slippery the interface between object and finger pad, the higher the GF at any given LF (1). There is a strong correlation between applied GF and moisture level at the finger pads. In particular, it was demonstrated that moisture regulation tends to stabilize the skin hydration toward the value that minimizes GF (66).

### Partial Slip Phase

The second phase, named “partial slip,” is a fast transitory state that begins when the object-finger contact areaprogressively changes with a very reproducible pattern, as showed by in vivo studies (63, 67–69) and mathematical models (70, 71). Initially, the outer part of the finger pad contact area begins to slide while the inner ellipse remains static. As the LF increases, the contact area gradually decreases and eventually detaches, leading to fully developed slippage (72). However, the dynamics of the pattern of transition may be influenced by many factors, such as skin hydration (72) and physical proprieties of fingertip ridges (73). The ratio of the no-slip area to the entire contact area (stick ratio, SR) has been proposed as a quantifier of the level of partial slip at the fingertip, from 1 (no slip) to 0 (full slip) (62, 69).

Critically, sensory information of “localized slips” due to the expansion of the partial slip area triggers GF adaptation well before the occurrence of full slip, at about half the minimum latency required for a voluntary response (2). At least two mechanisms could account for the detection of slippage during this phase. When the sliding surface has sharp irregularity (e.g., a small single protrusion of 4 μm high, 550 μm diameter) neighboring RA mechanoreceptors are activated sequentially as the surface moves, providing a reliable spatiotemporal code (74). Instead, when the sliding surface is smooth, with a low grip force value (0.2 N) subjects were not able to detect the slip (74), but when sufficient force (2N or more) was provided the fingertip deformation alone allowed slip detection (75). Localized slip responses were found in the majority of the FA1 and SA1 units, but rarely in FA2 units (2). FA1 units discharge during mechanical changes such as the local redistributions of the strain pattern (2) (Fig. 2D). These units are highly sensitive to transient deformation and low frequency vibration occurring during changes of skin indentation. The duration and intensity of FA1 afferents discharge reflects the duration and rate of the load force ramp increase (76). By contrast, they are rather insensitive to static skin deformation and very low frequency vibrations (76). SA1 units respond intensely during the lateral compression of the skin, and to skin indentation depth with an almost linear discharge (77). The combined response of SA1 (which transmit pressure feedback) and FA1 units (which transmit skin motion sensations) could efficiently code localized slips in a limited part of the contact area. Remarkably, all afferents fibers, except FA2, respond to the LF perturbations before the onset of the subject's corrective response, especially the FA1, which respond early enough to trigger the GF adaptive response. This evidence accounts for FA1 units capability to signal strain changes generated between the skin and the object (2), and to initiate the automatic increase of GF (2, 78). The highest density of FA1 receptors on the fingertip skin area accounts for their capability to initiate automatic modulation of GF (79). Interestingly, 30Hz vibration delivered from the outer to the inner part of the contact surface on the fingertip (therefore mimicking the physiological response) is able to elicit a “virtual” partial slip sensation and higher GF reflex (80). The skin hydration level seems to dramatically affect the dynamics of the contact from stick to slip phase, as well as during the stable grip (i.e., stuck phase). Subjects with naturally dry skin not only respond with a tighter grip to compensate for a reduced coefficient of friction, but also clearly overcompensate (i.e., the increment of GF is more than the necessary one to compensate a reduction of the coefficient of friction) (81). Further studies found also that the contact transition from adhesion to slip varied as a function of skin hydration. The greater the skin hydration level was, the further the contact was from slipping (72).

### Full Slip Phase

The third “full slip” phase occurs when the entire contact area slips. As the digits involved in the grasping and the surface of sliding object develop a relative velocity, asperities of both surfaces collide, creating high frequency mechanical perturbations (82). Fingertips are the most sensitive sites for vibrotactile detection; however, motion-related vibrations propagate to the hand and the forearm as well (83). Vibration threshold decreases with age everywhere but at the fingertips elders have the same ability of younger people to detect vibrations (84). The vibration frequency spectrum depends on several factors, such as the texture of the surface and the speed of the object slippage (83, 85, 86).

All units except SA2 exhibit responses during the full slip. However, while FA1 and SA1 initially discharge during localized slips, FA2 fibers (i.e., “Pacinian corpuscle” afferents), which have large receptive fields and are sensible to high frequency vibration, discharge preferentially during the full slip, and are almost silent before (2, 87) (Fig. 2D). As increasing speed produces a corresponding increase in both frequency and amplitude of vibrations (82, 83, 86), FA2 units may efficiently encode the slip speed, especially when the slipping surface is smooth. Indeed, the presence of irregularity like dots and ridges can affect the firing rate of the FA1 units (88). SA1 fibers, which respond preferentially to low frequency vibration (79, 89–91) are rather insensitive to changes in motion speed (92).

Vibration is an important cue for slip detection and its parameters, such as frequency or spatial propagation, are linked to the perception of slip. When sudden LF perturbations or vibrotactile stimuli were presented alone to an object steadily held, corrective motor actions occurred with a latency of 139ms and 229ms, respectively. However, the administration of anticipatory vibratory cues 50ms before perturbative loadings facilitated the GF adjustment by shortening the onset of motor responses of ~20ms (mean latency was reduced to 117ms)(93). When vibrotactile stimuli were presented at slip onset, or during its occurrence, the perceived duration of slip increased, whereas it decreased when they were presented early during the stuck phase (64). Moreover, when masking vibrations unrelated to slip are delivered, they impair the discrimination of slippage velocity (88). Vibratory parameters generated by different textures are related to the different latency in response to slippage occurrence, suggesting that vibratory cue generated by an accidental slip plays a rule in the grip modulation (94). The vibration due to the fingers-object sliding likely provides an efficient haptic cue to estimate their relative velocity, being the vibration power linked with the square root of the velocity assuming constant friction force (82, 94). Indeed, although the initial slip reflex is likely triggered by the increase of shear forces on the fingers before the object full slippage, the CNS may take advantage of vibration to further modulate additional grip force (94). Finally, in the absence of clearly detectable surface features (i.e., when managing a smooth object such as a glass of water), vibratory cues become particularly important to estimate the skin-object relative velocity (88).

### Slip Reactions: From Reflexes to the Voluntary Response

**Figure 3:**
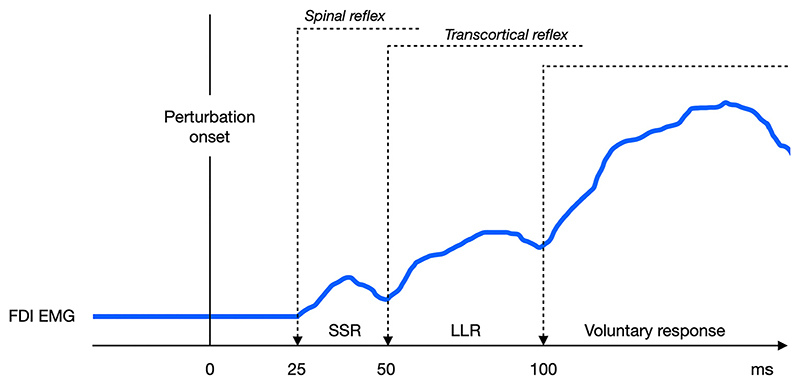
The evolution of the first dorsal interosseous (FDI) muscle EMG signal shows the stereotypical sequence of responses to a strong sudden perturbation, such as object pull (black continuous line). LLR, long-latency reflex; SLR, short-latency reflex.

The human nervous system counteracts unpredictable LF perturbations with a stereotypical sequence of activity in arm and hand muscles, starting from the simplest and rapid short-latency stretch reflex and ending with a more complex and delayed voluntary response (95–97) (Fig. 3).

The earlier compensatory muscle activity that can be seen as first EMG component is produced by the “short-latency reflex” (SLR), has an onset latency of ~25-50ms after the perturbation and it is generated by spinal circuits (78, 98). It is resistant to voluntary control and can be generated by either electrical stimulation of the digital nerves (99) or mechanical perturbations (78, 100).

The second component, termed “long-latency reflex” (LLR), has a latency of 50-70 ms after the perturbation. LLR has been frequency studies in regards of forearm muscles and is a highly sophisticated response: studies on healthy subjects and patients suggest that it originates from both spinal and supraspinal pathways circuits, including the primary motor cortex and reticular formation (101–104). LLR has contributions from different components that overlap in time. Indeed, one of its functional constituents appears to be resistant to voluntary control, similarly to SLR (97), whereas the other constituent shares features with the voluntary response since it is modulated by the intent of the subject or the predictability of perturbation (105, 106). Indeed, LLR is neither strictly automatic nor voluntary, as subjects instructed to ignore the perturbation continue to exhibit the reflex, but the response is larger if they try to resist to it (104).

Early somatosensory evoked potential studies showed a correlation between LLR elicited by muscle stretch and recorded cortical potentials (107–109). In monkeys, it has been shown that the cerebellum may mediate part of the LLR, via cerebello-thalamocortical projections that terminate within M1 (110). Indeed, the earlier cortical potential in response to a sudden perturbation consistently preceded the late EMG activity by 30–50 ms, suggesting a transcortical mechanism for LLR (108). Transcranial magnetic stimulation (TMS) studies also provided evidence that LLR is, at least in part, conducted along a transcortical pathway. EMG responses in the “flexor digitorum profundus” are greater when TMS stimuli are delivered on the motor cortex during the latter part of LLR (111). As the facilitatory effect persisted after the median and ulnar nerve blocks, muscle receptors contributed to the increased excitability of the motor cortex (111). A similar facilitation of motor evoked potentials in “thenar” muscles was found when TMS stimuli were delivered at intervals corresponding to the LLR (112).

Finally, pure voluntary reactions are generated at a more variable and longer delay (later than 100ms) after the perturbation, with the onset that depends mostly on the experimental condition (104). Voluntary responses require a highly distributed network, including premotor cortex and basal ganglia, that become active meanwhile faster spinal circuits continue to exert their contribution (113, 114). Contrary to SLR and LLR, voluntary reactions can be deliberately suppressed (104).

**Figure 4:**
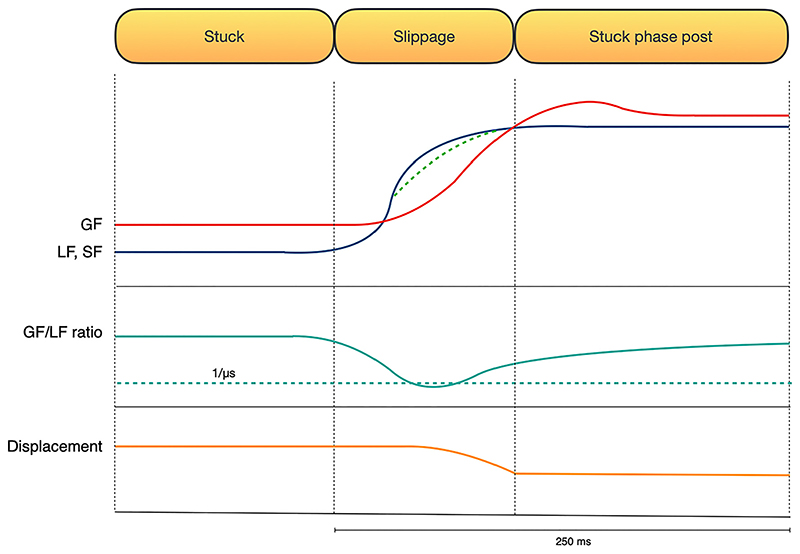
Relation between the grip force (GF, in red) shear and load force (SF and LF, in green and blue) when the slippage of the object is avoided. During the “slippage phase,” the LF/SF increase. In the case that the GF increases activating a compensatory response which overcomes LF, slippage is avoided. During the “stuck phase post slippage,” the GF is modulated to reestablish the optimal safety margin. The ratio between the GF and the LF is showed in green, the displacement (in orange) happens when that ratio value goes below the inverse of the coefficient of static friction (dotted green line).

Crucially, mechanoreceptors of the digits are the solely able to trigger SRL in the absence of forearm displacement (78). However, during mechanically less restricted conditions (115), or when cutaneous signals are unavailable or impaired (31), afferents from more proximal muscles, joints and tendons can also contribute to GF adjustments (31, 116, 117). These nondigital afferents respond more variably and with longer latencies (117). The magnitude of the GF adjustments varies at different load direction and hand orientation (118), depending on frictional anisotropies at the digit-object interface (119, 120), so that a similar safety margin against frictional slips is ensured (118). When the grip reaction is fast enough, to compensate the sudden increase of the LF/SF, the slippage of the object is avoided, and a new stuck phase starts (Fig. 4).

### Subcortical and Cortical Correlates of Slip Detection/Perception

Decoding of sensory inputs starts well before the cortical stages (121), and the afferent information is encoded at subcortical level by neurons of the cuneate nucleus (122, 123). Since each primary afferent has multiple synapses in the cuneate nucleus, mechanical stimuli in a specific skin region activate a large number of its neurons (124). Interestingly, the cuneate nucleus shows specific pattern of responses to different stimulation, including slip, with preferencial activation for certain stimulation types (121).

At a cortical level, various areas contribute to slip perception. Neurons in the primary somatosensory area (S1) are sensitive to the velocity of the moving stimulus, as showed using mechanical deformation of the skin (125), sequential stimulation of a fixed length of skin (126) and passive or active touch (127, 128). Speed-sensitive neurons were found predominantly in the S1 and S2 areas (129). Remarkably, the discharge patterns of these neurons have similarities with those seen at the level of primary afferents: their discharge rate increases gradually as a function of speed, and is sensitive to the texture surface (130). Not surprisingly, lesions of primary somatosensory cortex (S1) greatly impair the ability of monkeys to categorize the tactile velocity (131). In humans, tactile motion perception increases S1 activation (132–134), and the activation of multisensory areas such the middle temporal cortex (hMT + /V5) (134, 135) and parietal cortices (IPC) (133, 134). Repetitive transcranial magnetic stimulation (rTMS) applied on the hMT + /V5 interfered with tactile speed discrimination (136, 137), and double-pulse TMS on hMT + /V5 disrupted direction discrimination (138), suggesting a key role for those areas in motion processing. The cingulate motor area and the medial cerebellum are activated by both sudden loading (increase of LF) and sudden unloading (decrease of LF), whereas the primary motor cortex by sudden loading only. Likely, although M1 implemented corrective grip forces after the perturbation, both the cingulate motor area and the cerebellum participated in the sensorimotor representations of the fingertip forces (139)

As regard to the cerebellum, spontaneous slip of an object from a monkey hand produces a discharge in the majority of Purkinje cells, in the area of the anterior lobe which receives afferents from the hand (110,140). The majority of these neurons provide both preparatory and reflex responses; however, the slip of a handheld object has a maximal impact on cerebellar neurons at around ~40 ms (110,140). Given a conduction latency of 3–5 ms from the cerebellum to the motor cortex (141) and other 11ms between the motor cortex and the intrinsic hand muscles (142), these neurons seem to participate in reactions at 50–100 ms. However, to date, evidence in humans have not been provided.

Only few studies employed electroencephalography (EEG) to investigate cortical responses to predictable and unpredictable perturbations. Marked shape differences and increased amplitude in somatosensory evoked potentials (SEP) were found during perturbations with slip compared to trials without slip (143), suggesting that the neural coding is different when slip occurs. N58-P58 (or N54-P54) component shows higher amplitude when unpredictable perturbations occurred (95, 103), and likely represents the source of the sensorimotor cortex activity immediately preceding the LLR.

## Sensors and Haptic Devices to Convey Slip Feedback

To deliver slippage feedback, prostheses should be equipped with appropriate sensors to detect the slippage; whereas stimulation devices should be employed to deliver the information detected by these sensors to the remaining functional sensory system of the user.

For example, to measure SF during manipulation, diaphragm structures such as membranes (144) or cantilevers (145–147) have been designed to integrate silicon-based force sensors capable to measure their deformation. Polymer-based piezoelectric film sensors (e.g., PVDF: polyvinylidene fluoride) embedded in elastomeric materials, being able to record high frequency events, have been widely employed to detect the dynamics of incipient slip (148–151). For an indepth analysis see Romeo et al. (152).

Among the methods employed to provide tactile information to the intact sensory system of the amputee, sensory substitution techniques, such as vibro-tactile or electro-tactile stimulations (153–156), have been largely used, being low-power, unobtrusive and potentially embeddable within the socket. The sensations elicited by these stimulation techniques are far from being natural, as they exploit a sensory modality different than the one of the original sensory input. These sensory substitution techniques have been tested in unrealistic virtual environments and with control methods that are not applicable in a real scenario, for example, a robotic joystick to interact with virtual fragile objects or devices that, unlike those commonly used by amputees, are not myoelectric controlled (153–156). However, although the sensation elicited by vibrotactile stimulation is far from completely matching the input information, neurophysiological principles have been exploited to integrate it more easily in the user-prosthesis control loop.

For instance, the Discrete Event-driven Sensory Feedback Control (DESC) policy has been exploited to overcome vibro-tactile adaptation issue over time (157). It states that manipulation tasks are marked by discrete sensory events resulting from object contact, lift-off, etc. (42, 158). Accordingly, discrete vibro-tactile stimulations have been delivered at slip event to re-establish a stable grasp of an EMG-controlled robotic hand (52).

Modality-matched feedback, where the information is provided in the same modality as the sensory input, is more intuitive, allowing faster elaboration, lower cognitive load and shorter learning curve (34).

Noninvasive devices capable to provide modality-matched feedback of were successfully employed in prosthetic research: information on GF and LF during object manipulation have been delivered in patients that underwent target muscle reinnervation (TMR) (159, 160) through an ad hoc designed feedback device (39).

Moreover, several solutions employed in other research fields could be of interest: for example, good results in feeding back slippage sensation were obtained in laparoscopic surgery, by translating grasping forces with a cylindrical rotatory drum to the user’s thumb (161).

Tactile displays are further promising electromechanical devices to provide slippage sensation. By presenting distributed shear and normal forces, they reproduce the texture of an object surface on user’s skin, such as the finger pad. For instance, a device with the dimensions of a PC mouse could reproduce tactile sensations similar to moving edges on finger pad. It was implemented with a two-dimensional electromagnetic-linear motors to present LF in two directions, and a tactile display composed by an array of piezoelectric-actuators to reproduce distributed GF on the pad (162).

A system integrating a tactile display capable to provide distributed 30Hz vibration from the outer to the inner part of the finger pad contact area, coupled with a haptic device to provide combination of LF and GF solution, was proposed to reproduce the dynamics of the partial slip in virtual reality environments and teleoperation tasks. This system was able to generate a partial slippage sensation (80) by activating the Meissner’s corpuscles, which are crucial for slip detection.

Invasive methods, such as implanted neural interfaces, are deemed to have the potential to recover nearly all somatic sensations with a modality-matched feedback (34). Intraneural stimulation was delivered in the peripheral nerves of a transradial amputee and to healthy participants (through microneurography), who discriminated slippage and texture of different sliding surfaces thanks to an algorithm translating in neural code (i.e., spike-like signal) the data recorded by a fixed instrumented artificial finger (163). Such technique could be useful for the encoding of the full slip phase of different surfaces. Recently, a step beyond has been done to rely slip sensation in a transradial amputee. Electrical stimulations were injected into different nerve sites to elicit sequential sensations in different digits (i.e., the index and middle fingers) to reproduce the movement of an object between fingers (47). This solution enabled the control of slippage during manipulation tasks and allowed compensatory responses to the object slip (47).

Importantly, solutions capable to provide some close-to-natural sensations reduce abnormal phantom limb pain (164, 165) and promote the ability of the prosthesis to be perceived as a natural extension of the body (“embodiment”) (165–169). However, besides its promising possibilities, intra-neural interfaces require a surgical procedure which is not free from risks (34, 170).

Neural interfaces aim to restore biomimetic sensations, which is a very ambitious objective not completely achieved so far (171, 172). Indeed, the quality of the sensation perceived by individuals is often assimilated to a paresthesia, vibration, tapping or flutter on the skin. The path to improve perception goes through the enhancement of the selectivity of the stimulation. Novel neural interfaces should depolarize specific targeted fibers, while avoiding the activation of several bundles of afferents from many different types of cutaneous receptors all at once. In addition, a potential issue is the foreign.

## Insights From Physiological Basis

Several strategies can be followed to implement, on hand prosthesis, a system to avoid object slippage. These strategies range from completely automatic systems (low-level control), meaning that the prosthesis is autonomous for securing the object grasp, to solutions that restore sensory information to the prosthesis and to its owner, allowing the voluntary modulation of the hand-object interaction parameters by the user (high-level control). However, the extraction of sensory information is common to both low- and high-level solutions, and tomorrow prosthesis would not be able to do without one of those two control level.

To avoid object slippage in prosthetic user, one may be inspired by the physiology or think to non-biological-like solutions. In this section, we discuss possible solutions, focusing on the former approach. However, being inspired by the anatomical and functional principles of hand sensorimotor control loop managing slippage is an opportunity, never a constraint, and non-physiologically inspired out-of-the box solution which achieve the objective should be also considered.

Regarding completely automatic methods, a system can be implemented to ensure that GF/LF ratio always remains above the critical value to avoid object slippage. When the object is safety maintained in the hand (i.e., during the “stuck phase”), the absence of finger-object displacement despite changes in SF is mostly due to the coefficient of friction, which is largely influenced by the level of moisture. It may be envisaged the implementation of an artificial finger pad that integrates a microfluidic system capable to regulate its surface lubrication, mimicking the human finger pad. The automaticity of this behavior may be implanted employing finger pad built with deformable material. The release of the moisture could be activated or inhibited by means of changes of the shape of the pad because the applied force determines the status of the finger deformation during grasping. The level of the moisture should be timely regulated to maintain the coefficient of friction within an efficient range, as too much moisture could decrease the friction between skin and the object surface, leading to slippage instead of preventing it. The regulation of the moisture should be completely automatic, not involving the user. To reproduce the friction characteristics of the human hand, the physical features of glove of the robotic hand could also be different on basis of the covered areas; for example, the friction coefficient of the cover of the palm (or other areas not specialized for the grasp) could be made higher than that one of the finger pads by using different materials or superficial textures.

Another autonomous method to avoid the slippage consists of implementing a system capable to provide fast and automatic grip force adjustments whenever slippage is detected. In regards of human physiology, important cues can be gathered by the physiological timescale of the responses to slippage and by understanding which neural network is in charge of which. When slippage occurs, in a very fast transitory state (partial slip phase) the object-finger contact area progressively decreases. Given that partial slip responses trigger GF reflexes, the 25–50ms peak of the short-latency stretch reflex (SLR) and 50–70ms latency of the LLR set roughly the necessary margin of the reaction time that the low-level control embedded in the prosthetic system can take to adjust the forces and automatically avoid the object slippage. Therefore, such control system should work with a latency within 70ms. Automatic grip force adjustments can be implemented on each finger to be independently controlled according to the local coefficient of friction, which is particularly relevant for prostheses that allow more than two-fingers grasp (173). Another aspect to consider is that the sum of the forces exerted by the digits and the palm allows to maintain safe the object on the hand. Hence, force sensors should theoretically be integrated in each surface of the hand that can get in touch with the object to allow an algorithm reconstructing the forces exerted on the object during the grasp. Different friction coefficients may also require different spatial selectivity of sensors, located deeper in the palm to emulate the FA2 receptors and more superficially in the finger pads. In addition of being sufficiently fast, automatic grip force adjustments should not be too much above the force threshold required to avoid slippage, as too high GF may damage fragile objects. Additionally, in prosthetics, energy consumption matters, especially in transradial amputees when motors, gears, electronics and battery have to be all placed in the hand. Thus, the battery has little room and should not weigh too much and maintaining the generated GF at the minimum needed level could help battery consumption.

The opportunity to devote slippage control to an automatic system should be paralleled by a strategy to allow the volition of the user to take control over the automatic algorithm when they want to voluntary release an object. For example, to place a handheld object on a table, the user might perform a sustained action (e.g., opening hand movement for 2 s) that can be detected by EMG sensors and used as a trigger to turn down the automatic grip system.

In light of that, why do we not develop hand prostheses with automatic grip control always maintaining the GF/LF ratio above 1/μ_s_ or sticky fingertips that avoid the smallest slippage in any circumstance? Because the benefits of actively controlling slippage in a timely and efficient way go beyond its simple avoidance. Indeed, in daily activities, slippage can be voluntary modulated during the manipulation of objects. For example, when gently placing a handheld object on a table, passing a coin from one finger to another, or when fingers slide on the strings to play guitar or violin. In addition, slippage may have a strong emotional component, as when caressing a surface or in interpersonal contact. Therefore, an efficient and physiologically inspired control system should not only be always able to avoid the object slippage, but also permit an efficient slippage modulation, depending on the context, the goal, and the previous experience of the action performed. Not just avoiding, but smartly modulating slippage means to involve the user in the slippage control loop of the prosthesis, thus the readout of the sensors relative to slippage should be provided to both the prosthesis embedded control and to the user, which share the control of the prosthetic hand (shared control architecture) (35, 174). Such solution should include a low-level control loop, mainly responsible for the almost automatic grasp stability, and a high-level control loop, involving the user that exploits conscious sensory cues mostly for motor planning and correction.

Back to the physiology, the high-level control acts through the voluntary response (starting after 100ms) and through an intermediate long-latency reflex (LLR) which has a latency of ~70ms. Given that tactile information takes around 20ms to reach the somatosensory cortex (175), to implement artificially similar timing, the sensory stimulation should be delivered to the user in a time window within 50ms, which should include sensing, decoding and stimulating times.Several studies clearly showed that LLR has both automatic and voluntary components. Therefore, this physiological response represents an intermediate control level, which is not strictly automatic (as the SLR) nor completely voluntary (as voluntary responses). More importantly, LLR is modulated by the user intention and the nature of the task (104–106), so its level of automaticity can be changed. Such intermediate level of control should be implemented on a prosthesis to allow the precise setting of the degree of autonomy of the device.

An aspect of interest of shared control architecture would be the implementation of a switch between different settings or even a dynamic shared architecture, corresponding to favoring the high- or, vice versa, the low-level control loops, based on the tasks to be performed. In tasks having as major requirements quick and safe responses (i.e., holding harmful materials) the low-level control loop may increase its control; whereas complex manipulation tasks, where the user has to select if slide or not the object among the fingers, such as during shaking hands, could be managed by a high-level control loop.

For the replication of the physiology of motor control during learning, this may have also important implication for the acquisition of proficiency in prosthetic tasks. For example, beginner users could prefer a more autonomous modality, because this type of regulation will permit a more safety (although less voluntary) control, whereas expert users may prefer a more voluntary control (less autonomous) setting to reach a more efficient and pleasant motor control. The degree of autonomy of the device could be controlled by setting thresholds on the values of the interaction parameters; for example, selecting a low threshold relative to GF/LF ratio makes the device less controllable by the user during the tasks. Ideally, these thresholds should be dynamically and autonomously set by the embedded artificial intelligence. However, meanwhile the technology will not be ready for that, a simpler switch would allow to rapidly change between pre-settings of shared control based on the user intention or the task.

To reproduce the complex physiological tactile response to slippage, artificial sensors should mimic the nature and configuration of mechanoreceptors of the skin. Considering that both slow (i.e., SF and GF behavior) and rapid (i.e., stick and slip events and vibrations) dynamic changes are important to identify the slippage, the prosthetic device should be equipped with sensors with different frequency responses, working in parallel. In particular, the SF and GF values and their slow dynamic changes could provide information about the stability of the object in the stuck phase, whereas the detection of high dynamic change is important in the full slip phase. Lastly, for partial slip detection, both slow and rapid changes are fundamental. From a spatial selectivity point of view, a more superficial sensor is able to discern between very close stimuli, whereas the integration between them, which is needed to infer the unity of a complex stimulus (e.g., a manipulated object), is assured by deeper and less selective sensors. Such kind of distribution of mechanoreceptors could be reproduced by embedding sensors in elastomeric materials at different depth because sensors placed deeper are more suited to sense low frequencies, whereas high frequencies events happening at the surface are more attenuated. For instance, deep silicon-based force sensors are capable to measure the coarse forces applied on the skin, whereas vibration could be detected by polymer-based piezoelectric film sensors placed closer to the surface. The specific frequency to which a particular sensor is more sensible depends not only on the constitutive feature of the device, but also on the employed decoding and encoding algorithms and the relative hardware. This should be taken into account in sensor selection because it could increase the dimension and power-consumption of the prosthesis. Those algorithms allow to translate the information recorded by the sensing systems to an information usable by the stimulating transduction and are influenced by both the operating principles of the sensors and the stimulation devices; for example, silicon-based force sensors employ high-pass filters to focus on high frequency components which are fundamental for identifying slippage, whereas the physical operating principle of piezoelectric sensors intrinsically allows to sense only high frequencies.

As regards of noninvasive methods to deliver tactile sensation of slippage, a preliminary investigation on healthy subjects suggested that the stimulation of the finger pad by applying an external force and a 30-Hz vibration distributed from the outer to the inner part of the fingertip contact surface is the most effective to evoke partial slip responses (80). Moreover, systems constituted by a tactile display and actuators capable to provide SF on the contact surface can reproduce tactile sensations similar to those provided by a moving surfaces during full slip phase (162). To elicit phantom slippage sensations in amputees, stimulation should be delivered on the area of the skin where patients refer finger phantom sensations. However, the components of the device (i.e., cylindrical rotatory drum and tactile displays) are cumbersome, thus they cannot be easily integrated in prostheses for distal amputations (e.g., for transradial amputees). Interestingly, TMR patients may represent a unique opportunity to test this method: the stimulation device could be more easily embedded in the large socket and act on the larger skin area of the chest where the hand nerves are rerouted.

As regards the invasive stimulation, the method we previously developed (47) to reproduce the movement of an object on the skin could be further advanced to assess whether slip feedback can be induced from adjacent areas of the same finger, to provide more focused sensations. According to the actual state-of-the-art on implanted nerve electrodes, such ultrafine stimulation to elicit sensations on contiguous areas on a same finger pad is difficult to achieve. However, it can be hypothesized that a development of high-density electrodes with smaller active sites would allow to reach this goal, being able to elicit sensation on smaller adjacent zones (4-5mm of diameter).

## Conclusions

We reviewed the physiological correlates of slippage and three phases of slippage were identified. The stuck phase, where the user maintains the object stable in the hand; the partial slip phase, during which the slippage begins; and the full slip phase, when the object slips from the hand. The physiological mechanisms of the automatic grip reaction were used to speculate on a control architecture with different degrees of autonomy of the prosthetic device. In low-level control (mimicking SLR), the slippage response is managed by the automatic response of the device and in high-level control (mimicking the voluntary response) the GF reaction is actively managed by the user. Lastly, in the intermediate level of control (mimicking the LLR), the control is not strictly automatic nor voluntary, but it is shared between the device and the user. Depending on the performed task, an active manipulation of the parameters would initiate and optimize the grip response. This smart and flexible high-level control could be implemented within the artificial intelligence embedded in the prosthesis.

In light of the proposed three phases model and of the strengths and weaknesses of the strategies used so far, we provided insights on how to develop novel physiology-based prosthetic devices. In particular, given the importance of the moisture level of the human finger pad in the stability of the stuck phase, we proposed an artificial finger pad capable to regulate its surface lubrication. We also proposed a sensory device constituted by a multilevel architecture of different type of sensors, that would allow to detect the interaction events that characterize the slippage phases (i.e., low and high dynamic events), with higher and lower spatial resolution. Despite being well-conceived and physiologically inspired, those solutions are still far to be implemented in a commercial device, for their costs and their present technological immaturity. Todays’ amputees need simple dependable solution that can, even slightly, help support their manipulation abilities, because prostheses mostly miss any sensory feedback at all. Nevertheless, knowing how millennia of manipulation evolution shaped the human response system to slippage will be inspirational for the development of tomorrow hand replacement, aiming for a more natural and live hand.

## Supplementary Material

Appendix

## Figures and Tables

**Table 1:** Main characteristics of the mechanoreceptors of the hand

	Meissner's Corpuscles	Pacinian Corpuscles	Merkel Disks	Ruffini Endings
Type	FA1	FA2	SA1	SA2
Receptive field	Small, sharp borders	Large, blurred borders	Small, sharp borders	Large, blurred borders
Location	Superficial dermis	Dermis and subcutaneous	Basal epidermis	Dermis and subcutaneous
Receptive field area, mm^2^	22	Entire finger pad	9	60
Spatial acuity, mm	3	10 +	0.5	7 +
Innervation density on finger pad, /cm^2^	150	20	100	10
Frequency range, Hz	1–300	1–1,000	1–100	0–?
Main functions in slippage detection	Detect microscopic slip between the skin and an object held in the hand, signal sudden changes in LF force	Detect vibrations transmitted through objects in contact with the hand	Sensitivity to points, edges, and curvature	Sensitive to static force and motion direction, respond to remotely applied stretching of the skin

FA1, fast adapting type 1; FA2, fast adapting type 2; SA1, slow adapting type 1; SA2, slow adapting type 2. Refs. 9, 59–61.
