# Supplementary material for: Neurophysiology of slip sensation and grip reaction: insights for hand prosthesis control of slippage
Source: J Neurophysiol. Author manuscript; Available in PMC 2022 Jul 31. (PMC7613203; doi:10.1152/jn.00087.2021)
Supplement: Appendix [file EMS150770-supplement-Appendix.pdf]

APPENDIX

PLATFORM FOR SLIP INVESTIGATIONS

Slip perception has been investigated employing different platforms capable to simulate the interaction between the grasped object and the contacted skin, and to record the physical and neurophysiological features of the responses. Besides for identifying strengths and limits of previous studies, we reviewed the employed hardware because it can suggest solutions to be implemented in the prosthetic devices. Miniaturized version of the employed sensors can be embedded in the prosthesis to collect slippage information and of their actuators to provide haptic sensation of object slippage.

Two macro groups of devices have been employed to investigate the physiological mechanisms that lie behind slip perception: *1*) instrumented objects (hereafter called "passive platform") capable to record various parameters during the interaction with the hand, for example, the employed forces and movement during manipulation (66, 176–179); and *2*) mechatronic platforms (hereafter called "active platforms") capable to control the movements of the contact surface between the object and the fingertips, while monitoring the interaction forces and/or skin deformation (64, 94, 116, 180).

Passive platforms have the advantage to allow the user to grasp and manipulate the object in a more natural way; however, in such configuration, parameters of interaction can be only recorded and not controlled. These devices have often been employed while asking participants to perform activities such as pick-and-lift tasks with objects of different weights, or in point-to-point object holding tasks (143). Grasping an object between the pads of the thumb and the index finger ("pinch grasp") is the posture commonly employed in such experimental settings. Although force transducers monitor just the contact (i.e., GF) and shear forces (SF) (24), passive platforms may also integrate a load cell capable to record torques. In such way, it is possible to extract the points of application of forces exerted by the digits on the instrumented object, identifying (by using the torque values), the lever arm of the exerted force with respect to the sensor position. A passive platform may also be equipped with accelerometers to record its movements (2, 177). Additionally, different mechanisms have been refined over time to allow the change of the texture of instrumented objects (19, 181). The minimum weight and size of the embedded sensors, which cannot be decreased below a certain level, are the main limitation to the shape and features of the instrumented objects. To overcome this issue, instrumented gloves or thimbles have also been employed in place of instrumented objects (176); however, these solutions reduce the sensitivity of the hand and may limit its natural movements (176).

Active platforms are recommended for the investigation of a single parameter, while maintaining constant (or permitting a low variation of) the other features. For example, SF can be monitored while controlling GF and/or slipping speed. Active platforms are constituted by a moving object and, optionally, a support to fix the position and/or the direction of the digits which are in contact with the object. Usually, the moving object displaces along the LF direction and only the contact of one finger can be monitored, typically the index finger.

Active platforms employ electric or hydraulic linear or rotative actuators, for instance, a rotatory motor connected to a drum, externally equipped with different textures that could slide directly on the fingertip of the participant (182).

Robotic platforms with more than 1 degree of freedom (DoF) can provide the contact textures, while maintaining still the fingertip, changing the direction of the stress applied on it (i.e., the tangential force is composition of two different cartesian force components) (163, 180, 183). Active devices may also provide perturbations, such as vibrations and/or impulsive forces, while the moving object is in contact with the hand (95). In active platforms, the interaction forces can be easily recorded by placing a load cell on the moving object, whereas the position of the moving object and its derivates can be monitored by potentiometers or encoders placed on the actuator.

Optical systems made by a plate of smooth and transparent material (e.g., glass or plexiglass) and a high-frequency and high-resolution camera can be used in both passive and active platforms to acquire the images of the fingertip skin deformations (66, 74, 184).

Downloaded from journals.physiology.org/journal/jn (045.090.124.010) on July 14, 2022.

Moisture of the skin can be monitored by using capacitive sensors, more sensible to low levels of hydration, and conductance-based (or resistive) sensors which prefer high hydration (145, 185). Commercial devices (e.g., Corneometers as capacitive device and the Skicon-200 as device based on conductance) are cumbersome to be integrated in active platform, where the contact surface has to be moved. Alternative solutions are research prototypes consisting of plastic support with gold covered electrodes, connected in a 'comb' configuration (81) or a moisture resistive sensor integrated on transparent support to study the fingertip deformation (186).
